# Food Insecurity in Families With Children or Young People With Autism: A Systematic Review and Meta‐Analysis

**DOI:** 10.1111/nbu.70047

**Published:** 2026-02-17

**Authors:** Emma Tuschick, Jo Smith, Benjamin Harrison, Matthew Youngman, Emma L. Giles

**Affiliations:** ^1^ School of Health and Life Sciences Teesside University Middlesbrough UK; ^2^ Research and Development Team, Tees, Esk and Wear Valleys NHS Foundation Trust, Flatts Lane Centre Middlesbrough UK

**Keywords:** autism spectrum conditions, food insecurity, meta‐analyses, systematic review, young people

## Abstract

Food insecurity is frequently reported among families of children with autism spectrum conditions (ASC), yet there is limited evidence synthesising its prevalence and impact. This systematic review aimed to examine and meta‐analyse the existing literature on food insecurity in families of children and young people with ASC. A comprehensive search across nine databases identified 39 papers, of which 11 met the inclusion criteria. Studies were included if they involved autistic children or young people under the age of 25 (and/or their family members) and focused on food insecurity. Eligible studies were critically appraised, and data were synthesised using both narrative and meta‐analytic approaches. Meta‐analyses of nine studies estimated a pooled prevalence of food insecurity at 29% (SE: 5%; 95% CI: 17%–40%; *z* = 5.35, *p* < 0.001), which increased to 31% following adjustment for publication bias. The review also found that food insecurity worsened during the COVID‐19 pandemic, contributing to increased caregiver stress and disruptions in eating behaviours. This review demonstrates the high prevalence of food insecurity among families of children with ASC and the complex interplay of social, economic and behavioural challenges they face. Addressing food insecurity in autistic households requires policy responses that extend beyond financial aid to consider the sensory, behavioural and nutritional needs specific to ASC. Future research should adopt standardised measures and prioritise the development and evaluation of inclusive, tailored food support systems that reflect the lived experiences of neurodiverse families.

## 1 Introduction

Food insecurity can be defined as ‘lacking regular access to enough safe and nutritious food for normal growth and development and an active and healthy life’ (Food and Agriculture Organisation of the United Nations 2022). Food insecurity remains a pressing global issue, with more than 333 million people facing acute levels of food insecurity in 2023, when families are uncertain about when and how they will access their next meal (World Food Programme 2023). In the United Kingdom (UK) specifically, despite being one of the world's wealthiest nations, food insecurity is growing. In June 2023, 17% of households in the UK were food insecure, increasing by 8.8% from the previous year (Francis‐Devine et al. 2023). Moreover, during the period of 2022/23, the Trussell Trust, an organisation dedicated to addressing food insecurity through its network of foodbanks in the UK, distributed the largest number of three‐day emergency food parcels ever recorded (Francis‐Devine et al. 2023).

Food insecurity has profound effects beyond physical health, extending to mental health and overall well‐being. One worldwide review (Ejiohuo et al. 2024) revealed that both adult and child food insecurity were associated with heightened levels of parental stress, anxiety and depression, as well as increased fear in children. Moreover, parental depression was reported to be correlated with child food insecurity, emphasising the complex interaction between family dynamics and food security. Food insecurity clearly emerges as a multifaceted issue, amplifying the risk of malnutrition and obesity and exacerbating mental health challenges such as depression and stress (Baspakova et al. 2024). Evidence also consistently links food insecurity with poorer developmental, behavioural and mental health outcomes, including impaired cognitive development, increased emotional dysregulation and heightened stress within family systems (Gallegos et al. 2021; Shankar et al. 2017). These effects may be increased in autistic children who already experience elevated sensory, behavioural and regulatory challenges.

Autism spectrum condition (ASC), also commonly referred to as autism spectrum disorder (ASD), is characterised as a neurological and developmental condition impacting individuals' interactions with others, communication abilities, learning processes and behavioural patterns. While ASC can be diagnosed at any stage of life, it is termed a ‘developmental disorder’ due to its typical manifestation of traits within the initial 2 years of an individual's life (National Institute of Mental Health 2022). Approximately 1 in 100 children are diagnosed with ASC worldwide (World Health Organization 2023), with boys being four times more likely to be diagnosed compared with girls (Drake Institute 2024), although this is partly due to the influence of gender biases in diagnosis. Girls often mask their traits better than boys, and their presentation of traits may differ, leading health professionals to overlook ASC diagnoses in females (Drake Institute 2024).

An estimated 700 000 autistic individuals reside in the UK, accounting for more than one in 100 people (National Autistic Society 2024). They are reported to be 1.5 times more prone to experiencing food insecurity compared to the general population (Karpur et al. 2021). Diminished parental incomes coupled with escalating day‐to‐day expenses, as well as rising educational and healthcare costs, heighten the probability of food insecurity within the ASC population (Rogge and Janssen 2019; Mccall and Starr 2016). Households experiencing food insecurity frequently face challenges in maintaining consistent access to food, a situation heightened by atypical feeding behaviours commonly observed in children with ASC. This dynamic amplifies the risk of compromised physical and mental health and overall well‐being within the family unit (Hazzard et al. 2023). By ensuring adequate diet‐related support for this population, parents will have greater time and resources to support their child with other aspects such as communication and social comprehension (Karpur et al. 2021).

Beyond atypical feeding behaviours, children with ASC and their families face several additional pressures that increase their risk of food insecurity. Families often incur higher day‐to‐day costs, including expenses for healthcare appointments, therapies, special education, transport and adaptive equipment, which place them under sustained financial strain (Rogge and Janssen 2019; Grosse et al. 2021). Caregivers also report reduced employment flexibility, reduced working hours or withdrawal from the workforce entirely due to intensive caregiving responsibilities, which lowers household income and increases economic vulnerability (Mccall and Starr 2016; Ou et al. 2015). These financial pressures are increased by wider challenges, such as long waiting times for specialist services, limited respite provision and low levels of social support, which make it harder for families to manage their food budgets and maintain reliable access to suitable foods.

Although a number of large‐scale U.S. studies, such as those using National Survey of Children's Health (NSCH) data, have begun to examine food insecurity in households with autistic children, no comprehensive synthesis exists to evaluate the prevalence across diverse sources or to interpret how food insecurity intersects with feeding difficulties common in this population. This systematic review and meta‐analyses aimed to fill this gap by synthesising evidence from both quantitative and qualitative studies. Our research question was: What is the prevalence of food insecurity in families with children and young people with ASC, and what are their experiences of food‐related challenges?

## 2 Methods

This review is reported in line with Preferred Reporting Items for Systematic Reviews and Meta‐Analyses (PRISMA) 2020 reporting guidelines (Shamseer et al. 2015). The protocol for this systematic review was not registered with PROSPERO since it stemmed from a master's student's dissertation and has since been updated and searches re‐ran.

### 2.1 Search Strategy

Searches of all databases were conducted in line with the PICOS framework (Methley et al. 2014) for conducting literature searches for mixed methods synthesis. Population included children and young people with ASC below the age of 25 years and their family members; Intervention/exposure included food insecurity; Comparators included neurotypical children (not having, or not associated with a brain condition); and the outcomes included experiences of food insecurity described in cross‐sectional, cohort, qualitative or quantitative research. An example search strategy can be found in Appendix S1. Searches were initially run in June 2023 by MY and re‐run in December 2023 by ET and BH, and re‐run again in February 2025 by ET for updates. In total, eight databases were searched: Web of Science, Scopus, Medline, APA Psych, PsychInfo, CINAHL, PubMed and ASSIA. Google Scholar was also searched with the first 10 pages being downloaded, given that they would provide the most relevant outputs (Haddaway et al. 2015). All results were uploaded to Rayyan (Ouzzani et al. 2016), where duplicate records were removed prior to sifting.

### 2.2 Eligibility Criteria

A mixed‐methods systematic review approach was adopted to synthesise both prevalence estimates (quantitative data) and experiential accounts (qualitative data), given that both are essential to understanding not just how common food insecurity is, but how it manifests and impacts families' daily lives. Papers were included if they were primary research in the English language, published in a peer‐reviewed journal within the last 10 years, included an autistic child or young person below the age of 25 years and the exposure involved food insecurity. Papers were also included if the outcomes related to experiences, reports, prevalence, rates, incidence and risk of food insecurity. Studies were restricted to the past 10 years to ensure relevance to definitions of food insecurity and current autism diagnostic practices, both of which have changed substantially over the past decade. The age cut‐off of 25 years was selected to include adolescents and young adults who may still reside in the family home, reflecting ongoing caregiving roles and household‐level food dynamics (NHS 2024). A detailed list of all search terms used for this literature search is included in Appendix S1.

### 2.3 Study Selection and Data Management

Once the duplicates were removed, two reviewers (ET, BH) sifted titles and abstracts against the inclusion criteria and randomly selected 20% of each other's papers to review. The two reviewers had a 93.2% (Cohen's k: 0.85) agreement rate (almost perfect agreement rate in Kappa statistic), and no discrepancies were sent to a third reviewer. The papers identified as potentially relevant underwent the complete paper screening process. All papers were collected and stored on Microsoft Teams for assessment. One reviewer (ET) sifted through all full papers, while a second reviewer (BH) independently double‐screened 20% of them. A 90% (Cohen's k: 0.62) agreement rate between reviewers was observed, which translated to a substantial agreement rate when converted into Kappa statistic. Any disagreements at either stage were resolved by discussion.

### 2.4 Data Extraction

A Microsoft Excel spreadsheet was developed for data extraction by two reviewers (ET, BH) which captured: the authors, year of publication, country of study, the aim of the research, study design, methods, sample size, tool used to identify autism, any additional diagnoses or disabilities, questionnaire type used, food insecurity, participant demographics, outcome measures, key findings, recommendations and conclusions.

### 2.5 Assessment of Quality

Two reviewers (ET, BH) used the Appraisal Tool for Cross Sectional Studies (AXIS) (Downes et al. 2016) and the Mixed‐Methods Appraisal Tool (MMAT) (Hong et al. 2018) to assess the quality of included studies. Appraisal results informed the narrative synthesis and interpretation of the review findings. Studies meeting > 75% of the AXIS criteria were considered high methodological quality; those meeting 50%–74.9% were considered medium quality, and those meeting < 50% were considered low quality. Two different tools were used to suit the methodological designs of the included studies: AXIS was applied to assess quantitative cross‐sectional studies, while MMAT was used for the single qualitative study, ensuring each study was appraised using a tool appropriate to its design.

### 2.6 Analysis

The data were reviewed by a quantitative researcher with experience of meta‐analysis (JS), who indicated that meta‐analysis could be undertaken. The Meta‐Essentials Workbooks for Meta‐analysis version 1.5 (Suurmond et al. 2017) was employed in conducting the meta‐analysis. Prevalence data on food insecurity, encompassing both percentages and means, were extracted from each included study to be incorporated into the meta‐analysis. An overall point estimate was calculated to understand the combined effect size across all included studies. As data for other variables was insufficient or collected in multiple ways, narrative synthesis was used for other quantitative variables and content analysis for qualitative data (Krippendorff 2018). Anticipating high heterogeneity within the data, an examination of food insecurity was conducted utilising a narrative synthesis approach (Ryan 2013). The outcomes were subsequently organised and showcased in the form of a frequency table (Table 1). Additionally, the studies excluded from the meta‐analysis were subjected to narrative synthesis (Popay et al. 2006) to provide a comprehensive understanding of their results.

**TABLE 1:** Summary of Included Studies.

Authors, date and country	Study design and population	Variables and measurement tools	Other mental illnesses/non‐ASD comparators	Key findings	Quality assessment tool and score
Anderson et al. (2023), USA	Design: Cross‐sectional data analysis Sample size: *n* = 12 143 Diagnoses: ASD (self‐reported) Gender: Unreported Age: 3–17 years Ethnicity: White (39.5%); Black (20.9%), Other (7.9%), Hispanic (31.7%)	Food insecurity: National Survey of Children's Health (NSCH) Feeding behaviours: Unreported	Other special care needs and non‐ASD	Prevalence of food insecurity: ASD children experienced significantly greater levels of general hardship than those of children with and without other SHCN (40.0% vs. 28.9% and 19.6%, respectively); however, they were not significantly more likely to receive cash assistance than either comparison group. ASD children were also more likely than children with no SHCN to be food insecure (14.0% vs. 7.9%) despite having higher rates of SNAP participation (54.0% vs. 44.0%)	Tool: AXIS Score: 20/20 Percentage: 100%
Brochier et al. (2023), USA	Design: Cross‐sectional data analysis Sample size: *n* = 1321 Diagnoses: ASD (self‐reported) Gender: 77.9% male Age: 0–17 years Ethnicity: White (43.1%) Hispanic (34%) Black (14.4%) Other (4.8%) Asian (3.7%)	Food insecurity: NSCH Feeding behaviours: Unreported	None	Prevalence of food insecurity: Food insecurity was significantly associated with higher disease prevalence and severity for ASD (aOR: 2.1; 95% CI: 1.5–2.7). Caregiver underemployment, low social support, and discrimination were significantly associated with higher disease prevalence of ASD. For each additional social risk factor a child was exposed to, their odds of having each condition increased: ASD (aOR: 1.4, 95% CI: [1.3, 1.5])	Tool: AXIS Score: 20/20 Percentage: 100%
Dhillon‐Burrows et al. (2023), England	Design: Interviews Sample size: *n* = 15 Diagnoses: ASD (self‐reported) Gender: 8 males, 6 females Age: 6–16 years Ethnicity: White British (*n* = 13), White Irish (*n* = 1) White and Black Caribbean (*n* = 1)	Food insecurity: Interviews Feeding behaviours: Interviews	None	Prevalence of feeding behaviours/food insecurity: Eating behaviours in autistic children were both positively and negatively affected by the COVID‐19 lockdown. Whilst all parents felt under pressure with extra care responsibilities, some described less concern over food intake during the first lockdown because they were able to support their child's mealtimes and have new food experiences. By the end of the lockdowns, many parents reported worsening of eating behaviours, with children more likely to have a restricted diet, and eating more snack foods. Food shortages also triggered additional stress with parents unable to access their child's preferred food	Tool: MMAT Score: 7/7 Percentage: 100%
Karpur et al. (2022), USA	Design: Cross‐sectional data analysis Sample size: *n* = 1515 Diagnoses: ASD (self‐reported) Gender: Unreported Age: Unreported Ethnicity: Unreported	Food insecurity: Autism Speaks' Food Insecurity Survey (AFIS) & Household Pulse Survey (HPS) Feeding behaviours: Unreported	Non‐ASD	Prevalence of food insecurity: After adjusting for background differences, households of children on the autism spectrum in the ASFIS were about four times more likely to be food insecure than households in the general population contained in the HPS data (OR 1/4 3.7; 95% CI: 3.1e4.4)	Tool: AXIS Score: 19/20 Percentage: 95%
Karpur et al. (2021), USA	Design: Cross‐sectional data analysis Sample size: *n* = 2238 Diagnoses: ASD (self‐reported) Gender: Unreported Age: 0–17 years Ethnicity: Unreported	Food insecurity: NSCH & National Survey of Children with Special Health Care Needs (NSCHCN) Feeding behaviours: Unreported	Intellectual disabilities	Prevalence of food insecurity: A substantially higher proportion of children with ASD + ID were food insecure (44%), followed by children with ASD only (40%), children with other disabilities (33%), and those with no disabilities (20%). Households of children with ASD + ID were about two times more likely to be food insecure than the households of children without disabilities. Further, the households of children with ASD were 1.5 times more likely, and those with other disabilities were 1.3 times more likely to be food insecure than the households of children without disabilities	Tool: AXIS Score: 18/20 Percentage: 90%
Kim and Kwon (2022), USA	Design: Cross‐sectional data analysis Sample size: *n* = 529 Diagnoses: ASD (self‐reported) Gender: 83.2% male Age: 10–17 years Ethnicity: Hispanic (38.34%), non‐Hispanic white (43.89%), non‐Hispanic black (11.90%), non‐Hispanic Asian (1.36%), and others (4.50%)	Food insecurity: Likert Scale Feeding behaviours: Likert Scale	None	Prevalence of feeding behaviours/food insecurity: Two‐parent households, less healthy parents and households, households with smokers, poor sleep quality, and greater participation in organized activities were associated with a higher likelihood of overweight in children with ASD (all *p* < 0.05). In terms of obesogenic factors, children who slept well had statistically significantly lower odds of being overweight than those who did not receive adequate sleep [OR = 0.38, 95% confidence interval (CI) = 0.19–0.71]. According to parenting capacity, children from two‐parent households (OR = 5.70, 95% CI = 1.83–17.79) were more likely to be overweight than children from any other family structure (e.g., one parent, no parents, etc.). In terms of community and school activities, children who had participated in more organized activities after school or on weekends over the past 12 months were more likely to be overweight than those who had not (OR = 2.74, 95% CI = 1.35–5.55)	Tool: AXIS Score: 20/20 Percentage: 100%
Magaña et al. (2023), USA	Design: Cross‐sectional data analysis Sample size: *n* = 124 Diagnoses: ASD (self‐reported) Gender: 100% male Age: 9–10 years Ethnicity: Unknown	Food insecurity: Individual socioeconomic position (SEP) Feeding behaviours: Unreported	None	Prevalence of food insecurity: The racial/ethnic distribution was: 17.1% Black, 14.6% Latino, and 68.3% White; average age was 10 years. Both Black (PR 2.57, 95% CI: 1.26–5.26) and Latino boys (PR 2.08, 95% CI: 1.08–4.03) with ASD were more likely to be obese than their White peers. While there were significant differences in some social determinants of health by race/ethnicity, only food insecurity mediated associations between race/ethnicity (Black vs. White) and obesity	Tool: AXIS Score: 16/20 Percentage: 80%
Panjwani et al. (2021a), USA	Design: Cross‐sectional survey Sample size: *n* = 200 Diagnosis: ASD (self‐reported) Gender: 76% male Age: 2–17 years Ethnicity: Non‐Hispanic white (62%), 38% unreported	Food insecurity: Validated two‐item screening tool (22 hager) Feeding behaviours: Unreported	None	Prevalence of food insecurity: Food insecurity pre: OR 1.7, 95% CI = 0.96–3 and *p* 0.01 and post COVID: OR 2.6, 95% CI = 1.45–4.67 and < *p* 0.001. Prevalence of feeding behaviours: Shelter regulations significantly contributed to adverse eating behaviours (OR: 2.30; 95% CI: 1.12–4.72, *p* = 0.03)	Tool: AXIS Score: 17/20 Percentage: 85%
Panjwani et al. (2021b), USA	Design: Cross‐sectional survey Sample size: *n* = 200 Diagnoses: ASD (self‐reported) Gender: 76.1% male Age: 2–17 years Ethnicity: White (62.1%), Black (7.2%), Hispanic (13.9%), mixed (9.7%) and other (7.2%)	Food insecurity: Self‐ created survey Feeding behaviours: Self‐ created survey	None	Prevalence of feeding behaviours/food insecurity: A majority of respondents reported a moderate‐to‐large impact on the child's overall behavior (74%) due to COVID‐19. Stratifying by income level and food security status revealed disparities in the impact on overall behavior and most specific behaviors. Compared to a household income ≥ $100 K, an income < $50 K was associated with an increased risk of moderate‐to‐large impact on the child's overall behavior (odds ratio (OR): 4.07, 95% CI: 1.60, 10.38). Food insecurity also significantly impacted this risk, even after adjusting for potential confounding factors (OR: 3.31, 95% CI: 1.13, 9.66)	Tool: AXIS Score: 14/20 Percentage: 70%
Tahech et al. (2023), USA	Design: Cross‐sectional data analysis Sample size: *n* = 59 725 (*n* = 1702 ASD) Diagnoses: ASD (self‐reported) Gender: 79% male Age: Mean 10.44 years Ethnicity: White (47.90%) Black (15.35%), Hispanic (27.31), non hispanic (9.44%)	Food insecurity: NSCH Feeding behaviours: National Survey of Children's Health	Non‐ASD	Prevalence of feeding behaviours/food insecurity: A greater percentage of parents of children with ASD reported weight‐related concerns about their child (*p* < 0.001), food insecurity (*p* < 0.001), and fewer family meals together (*p* = 0.04) compared to parents of NT youth. Results from the regression analysis revealed that the odds of weight concerns for youth with ASD were 2.29 times (95% CI = 1.62–3.25) the odds of weight concerns for NT youth	Tool: AXIS Score: 18/20 Percentage: 90%
Tanner et al. (2018), USA	Design: Cross sectional questionnaire Sample size: *n* = 35 Diagnosis: ASD (self‐reported) Gender: 32 males Age: 4–10 years Ethnicity: White (*n* = 22), Non‐White (*n* = 13)	Food insecurity: Validated two‐item screening tool (22 hager) Feeding behaviours: BAMBIC	None	Prevalence of food insecurity: Selective eating group: food secure (*n* = 13) and insecure (*n* = 4). Non‐selective eating group: food secure (*n* = 12), insecure (*n* = 6). Prevalence of feeding behaviours: Selective eating group ate fewer total foods (*p* < 0.001). Selective eating group had a higher rate of food refusal (*p* < 0.001). Significant relationship between the Food Refusal subscale and the Limited Variety, *r* = 0.486, *p* < 0.01 scale of the BAMBIC. Significant relationship between SSP Taste/Smell Sensitivity scores and total foods eaten, *r* = 0.363, *p* < 0.05. Significant relationships between SSP Taste/Smell Sensitivity scores and Food Refusal, *r* = –0.419, *p* < 0.01, scores on the BAMBIC	Tool: AXIS Score: 16/20 Percentage: 80%

## 3 Results

**FIGURE 1:**
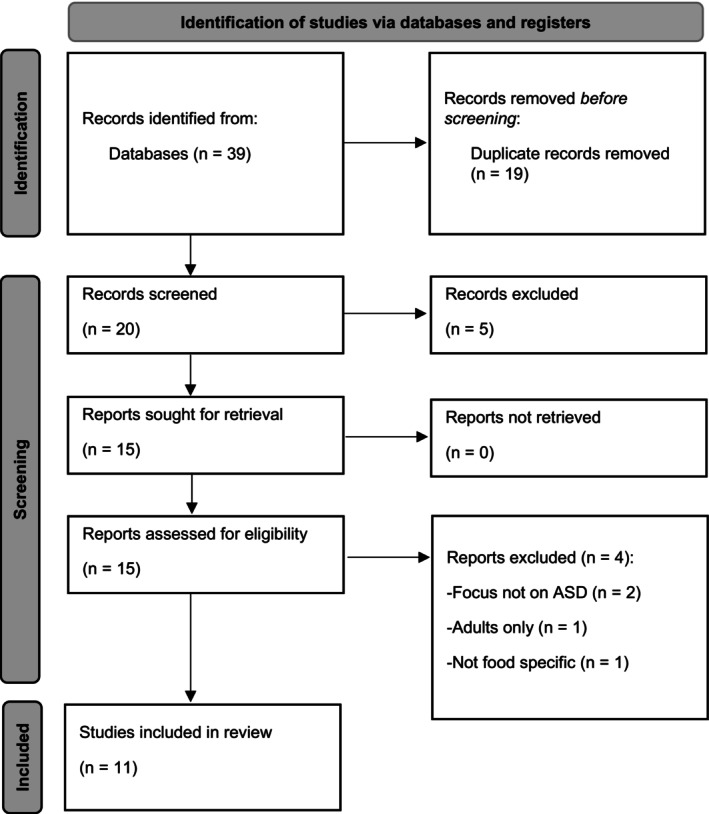
PRISMA diagram. Page et al. (2021).

The PRISMA flow diagram is shown in Figure 1. The initial searches identified 39 records. After de‐duplication (*n* = 19 total removed) and title and abstract sifting (*n* = 5 removed), 15 full papers were assessed. In total, 11 papers met the inclusion criteria and were included in the review. The articles were published between 2015 and 2023. The reasons for exclusions are documented in Figure 1.

### 3.1 Study Characteristics

The characteristics from the 11 included papers are presented in Table 1. Ten papers (91%) used a quantitative design (Anderson et al. 2023; Brochier et al. 2023; Karpur et al. 2021, 2022; Kim and Kwon 2022; Magaña et al. 2023; Panjwani et al. 2021a, 2021b; Tahech et al. 2023; Tanner et al. 2018) and one paper (9%) used a qualitative design (Dhillon‐Burrows et al. 2023). Four papers (36%) included comparators; ASD and non‐ASD (*n* = 2) (Tahech et al. 2023; Karpur et al. 2022), ASD and intellectual disability (ID) (*n* = 1) (Karpur et al. 2021), and ASD, special care needs and non‐ASC (*n* = 1) (Anderson et al. 2023). Ten papers (91%) reported on research from the US (Anderson et al. 2023; Brochier et al. 2023; Karpur et al. 2021, 2022; Kim and Kwon 2022; Magaña et al. 2023; Panjwani et al. 2021b; Tahech et al. 2023; Tanner et al. 2018) and one (9%) from England (Dhillon‐Burrows et al. 2023).

### 3.2 Participant Characteristics

The total sample size was 78 045. The mean percentage of males across all papers was approximately 80%, and the ages ranged between 0 and 17 years. Eight papers (Anderson et al. 2023; Brochier et al. 2023; Dhillon‐Burrows et al. 2023; Kim and Kwon 2022; Panjwani et al. 2021b, 2021a; Tahech et al. 2023; Tanner et al. 2018) reported participant ethnicity; mean percentage for White/Caucasian participants was 58% and Black participants was 18%.

### 3.3 Autism Diagnosis

The majority of studies used self‐reporting to identify an autism ‘diagnosis’ (*n* = 10) (Tanner et al. 2018; Tahech et al. 2023; Panjwani et al. 2021a, 2021b; Magaña et al. 2023; Kim and Kwon 2022; Karpur et al. 2021, 2022; Brochier et al. 2023; Anderson et al. 2023), with one study only including participants with a formal diagnosis of autism (Dhillon‐Burrows et al. 2023). Additionally, none of the included studies specified autism severity levels or functional support needs, limiting insights into whether food insecurity differs by severity of ASC.

### 3.4 Food Insecurity

Five papers looked specifically at food insecurity and six papers looked at both feeding behaviours and food insecurity. The most commonly used questionnaires to measure these included the National Survey of Children's Health (NSCH) (108) (*n* = 4) (Anderson et al. 2023; Brochier et al. 2023; Karpur et al. 2021; Tahech et al. 2023), 22 Hager (109) (*n* = 2) (Panjwani et al. 2021b; Tanner et al. 2018), Autism Speaks' Food Insecurity Survey (AFIS) (*n* = 1) (Karpur et al. 2022), Household Pulse Survey (HPS) (*n* = 1) (Karpur et al. 2022), National Survey of Children with Special Health Care Needs (NSCHCN) (*n* = 1) (Karpur et al. 2021), Likert Scale (*n* = 1) (Kim and Kwon 2022), Individual socioeconomic position (SEP) (*n* = 1) (Magaña et al. 2023), and a self‐created survey (*n* = 1) (Panjwani et al. 2021a). Other methods included semi‐structured interviews (*n* = 1) (Dhillon‐Burrows et al. 2023).

### 3.5 Methodological Quality

The critical appraisal scores for each study are reported in Table 1. For the Appraisal Tool for Cross‐Sectional Studies (AXIS) (Downes et al. 2016), all studies were of medium to high quality, meeting at least 65% of the AXIS criteria (< 50% indicates low quality, 50%–69% indicates medium quality, and > 70% indicates high quality). One study scored 70% (Panjwani et al. 2021a), two studies scored 80% (Magaña et al. 2023; Tanner et al. 2018), one study scored 85% (Panjwani et al. 2021b), two studies scored 90% (Karpur et al. 2021; Tahech et al. 2023), one study scored 95% (Karpur et al. 2022) and three studies scored 100% (Anderson et al. 2023; Brochier et al. 2023; Kim and Kwon 2022).

For the Mixed‐Methods Appraisal Tool (MMAT) (Hong et al. 2018), the study scored 100% (> 75% indicates a higher quality) (Dhillon‐Burrows et al. 2023).

### 3.6 Meta‐Analysis

For the meta‐analysis, 15 outcomes, that focused on food insecurity, from nine studies were included (Anderson et al. 2023; Brochier et al. 2023; Karpur et al. 2021, 2022; Kim and Kwon 2022; Magaña et al. 2023; Panjwani et al. 2021b, 2021a; Tanner et al. 2018). We could not analyse any papers that looked at both feeding and food insecurity together because no included studies reported quantitative effect estimates (e.g., correlations, odds ratios or comparable prevalence data) that linked food insecurity and feeding behaviours within the same analytic model. The meta‐analysis followed standard methodological guidance recommending a minimum of 10 outcomes to ensure sufficient statistical power and stability of pooled estimates (Deeks et al. 2024). Including 10 or more outcomes enables more reliable estimation of between‐study variance, supports meaningful assessment of heterogeneity and strengthens the interpretability of pooled prevalence estimates for informing clinical practice and policy. No further meta‐analyses could be undertaken due to heterogeneity across the data (including differing sample sizes, genders and ages) and measurement tools used.

#### 3.6.1 Unadjusted Meta‐Analysis

The combined effect size for the 15 outcomes included in the meta‐analysis for food insecurity was 29% (Standard Error: 5%, 95% Confidence Interval: 17%–40%, *z* = 5.35, *p* < 0.000). This means the unadjusted prevalence of food insecurity in the combined sample of participants with ASC was 29%. The heterogeneity of the studies was considerable (*I* ^2^ = 99.54%, *p* < 0.000, *n* = 16).

**FIGURE 2:**
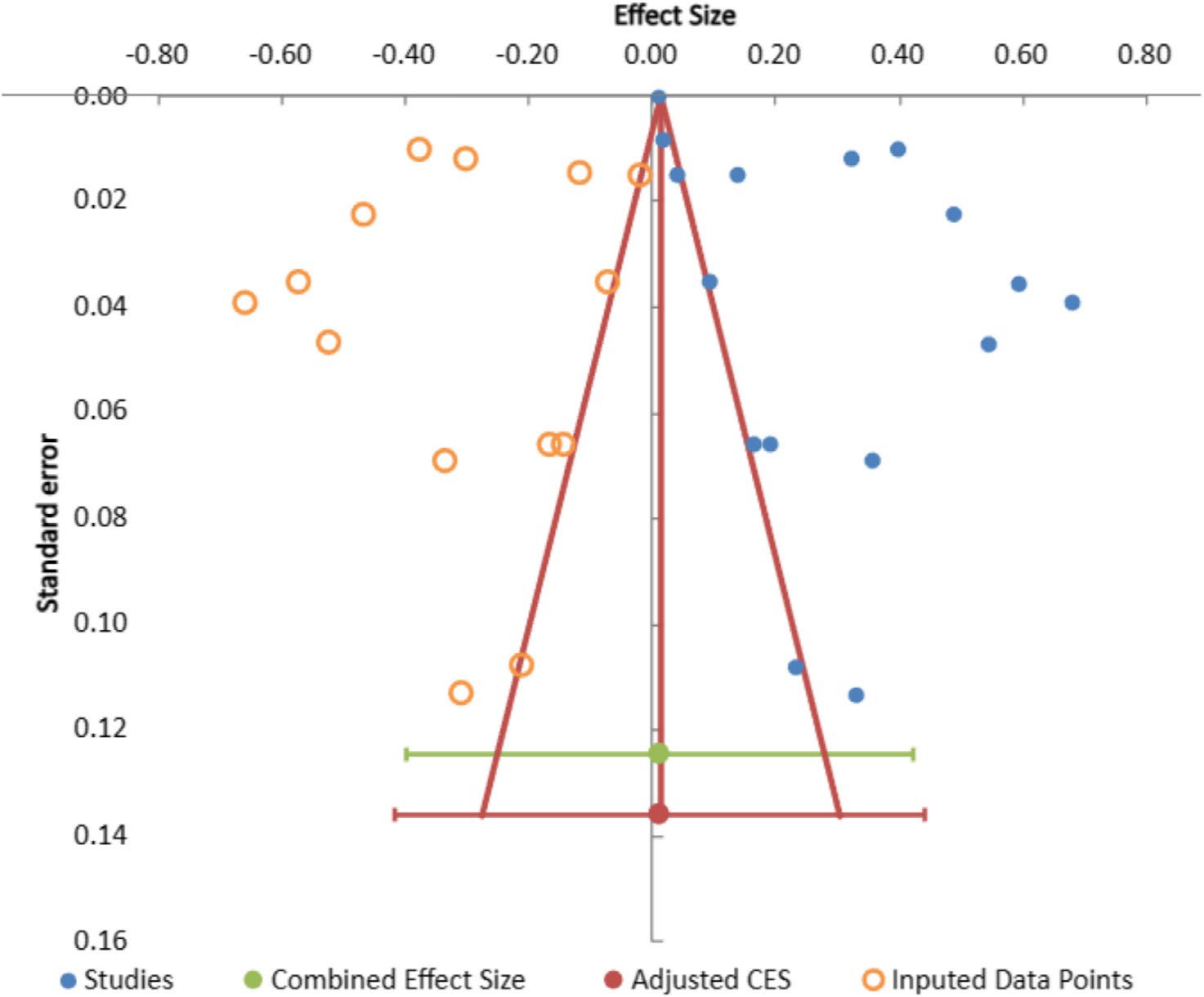
Funnel Plot for unadjusted analysis.

The Egger Regression test indicated significant publication bias (*p* = 0.002), due to one study having a very large sample size with unusually low prevalence rates (Tahech et al. 2023) compared to all other studies, which may have been driving this bias (see Figure 1, funnel plot). To assess the influence of this outlier, we conducted a sensitivity analysis excluding this study (Figure 2).

#### 3.6.2 Adjusted Meta‐Analysis (Leave One Out Analysis)

**FIGURE 3:**
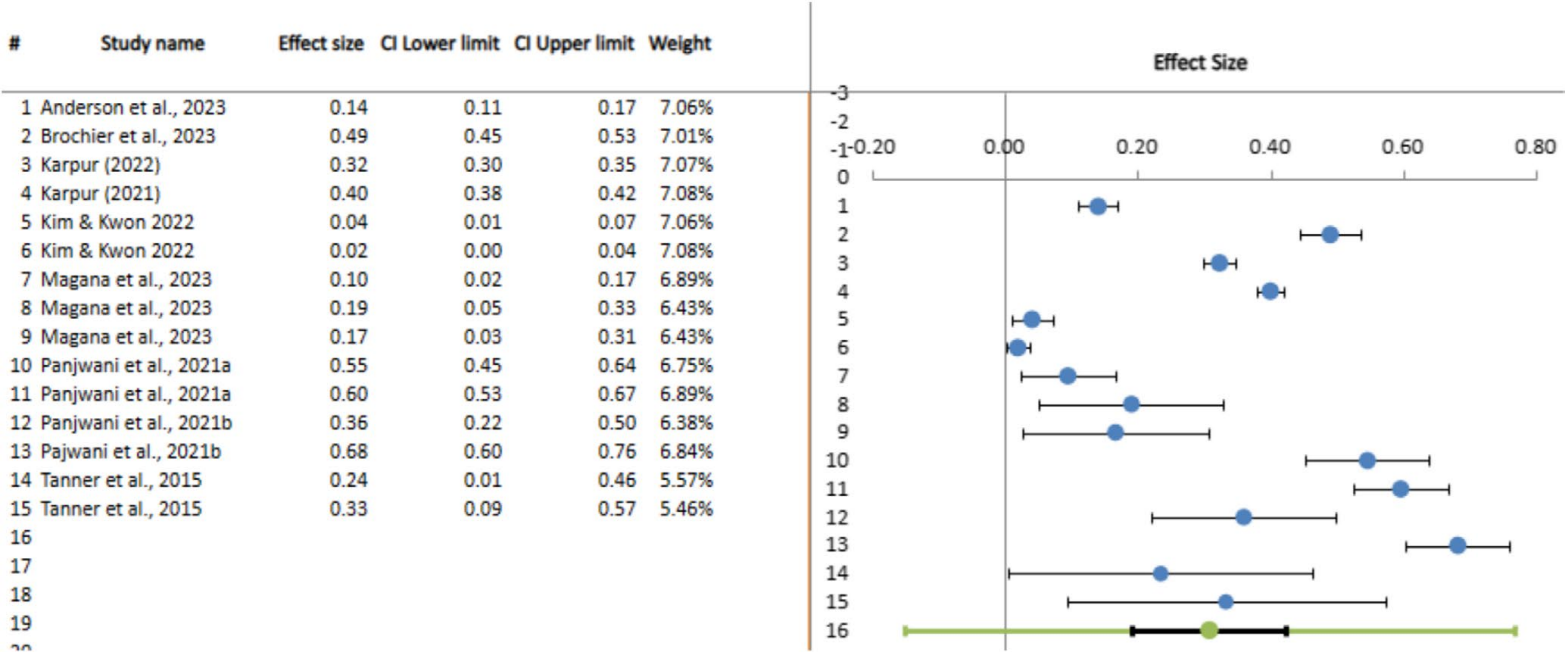
Forest plot for food insecurity prevalence for participants with ASC.

The combined effect size for the 15 outcomes included in the meta‐analysis was 31% (Standard Error: 5%, 95% Confidence Interval: 19%–42%, *z* = 5.72, *p* < 0.000). This means the adjusted prevalence of food insecurity in the combined sample of participants with ASC was 31%. The heterogeneity of the studies was considerable (*I* ^2^ = 99.09%, *p* < 0.000, *n* = 15). Furthermore, the included studies used different tools to measure food insecurity and ASC, which reduces the reliability of the effect size. The Forest Plot for the prevalence meta‐analysis is shown in Figure 3.

**FIGURE 4:**
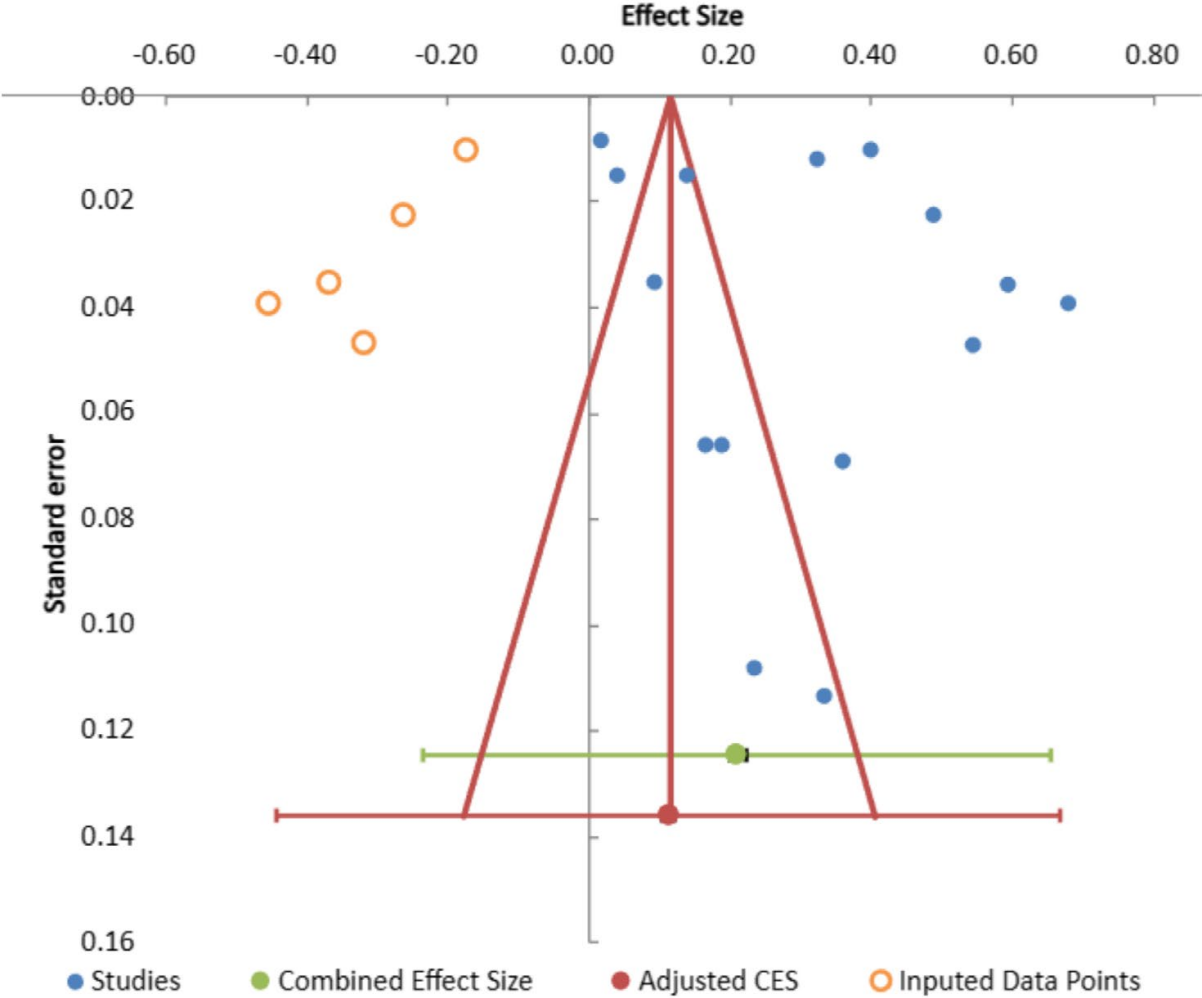
Funnel Plot for effect size against standard error for prevalence of food insecurity.

Figure 4 shows a funnel plot of effect size against standard error. There is considerable heterogeneity in the studies, as shown by the points outside of the lines. Furthermore, the Egger Regression test was not significant for publication bias (*p* = 0.252). Comparator analysis was not conducted due to insufficient data, with three studies comparing to children without ASC, two studies comparing against other disabilities, and five studies comparing within groups of children with ASC.

### 3.7 Narrative Synthesis

Eleven studies reported food insecurity data (Anderson et al. 2023; Brochier et al. 2023; Karpur et al. 2021, 2022; Kim and Kwon 2022; Magaña et al. 2023; Panjwani et al. 2021b, 2021a; Tanner et al. 2018; Dhillon‐Burrows et al. 2023; Tahech et al. 2023).

Tanner et al. (2018) reported that thirteen participants (76.5%) were food secure and four (23.5%) were insecure in the selective eating group, compared to twelve participants (66.7%) were food secure and six (33.3%) were insecure in the non‐selective eating group. While limited in number, these findings point to a potential relationship between atypical feeding behaviours, such as selective eating, and heightened risk of food insecurity in ASC households. Anderson et al. (2023) (40.0% vs. 28.9% and 19.6%, respectively), Karpur et al. (2021) (OR = 3.7; 95% CI: 3.1–4.4), Karpur et al. (2022) (40% vs. 33%), Panjwani et al. (2021a) (OR: 3.31, 95% CI: 1.13, 9.66) and Tahech et al. (2023) (*p* < 0.001) highlighted higher rates of food insecurity and material hardship among households of children with ASC compared to the general population or other groups with Special Health Care Needs (SHCN). This includes limited access to cash assistance, Supplemental Nutrition Assistance Program (SNAP) and higher levels of general hardship.

Three studies (Karpur et al. 2021, 2022; Brochier et al. 2023) identified associations between food insecurity, material hardship and social determinants such as caregiver underemployment, low social support, discrimination and household income. These social factors contributed to higher disease prevalence, severity and behavioural challenges in children with ASC.

External factors also played a role. Panjwani et al. (2021b) reported an increase in food insecurity pre‐ and post‐lockdown regulations during the COVID‐19 pandemic in the USA (Pre: OR = 1.7, 95% CI = 0.96–3, *p* = 0.07; Post: OR = 2.6, 95% CI = 1.45–4.67, *p* < 0.001). Similarly, Panjwani et al. (2021a) and Dhillon‐Burrows et al. (2023) found that lockdown measures negatively impacted eating behaviours and overall well‐being of children with ASC (OR: 4.07, 95% CI: 1.60, 10.38), exacerbating existing challenges and increasing stress for caregivers.

Disparities in obesity rates among children with ASC based on racial/ethnic backgrounds were also observed by Magaña et al. (2023) with Black and Latino boys with ASC being more likely to have obesity compared to their White counterparts. Food insecurity was identified as a mediator of these associations.

## 4 Discussion and Implications

This systematic review and meta‐analysis aimed to identify the prevalence of food insecurity in families of children and young people with ASC and to explore their experiences of food‐related challenges.

The meta‐analysis revealed an overall unadjusted rate of 29%, increasing to 31% after the exclusion of one study following a sensitivity analysis. These findings suggest that nearly one in three children with ASC live in food‐insecure households (31% adjusted prevalence), substantially higher than population‐level averages 6.5% in high‐income countries (Gatton and Gallegos 2023). However, substantial heterogeneity (*I* ^2^ > 99%) and evidence of publication bias limit the generalisability of these findings and point to methodological inconsistencies across studies. These issues reflect challenges commonly observed in public health and disability research, where varied measurement tools and study designs reduce comparability and confidence in pooled estimates (Scaglioni et al. 2018; Teasdale et al. 2019).

Additionally, it is notable that 91% of the included studies were conducted in the United States, which limits global generalisability and may reflect region‐specific systems of healthcare, education and welfare support. Furthermore, the gender imbalance in study samples, where approximately 80% were male, reflects longstanding diagnostic biases in ASC research. Girls may be underdiagnosed due to gendered symptom presentation and masking behaviours, which could affect both prevalence estimates and representation in food insecurity research.

Beyond the statistical considerations, the high pooled prevalence has meaningful real‐world consequences. Food insecurity in autistic households often co‐occurs with elevated support needs (Christopher et al. 2023), higher caregiving demands (Warreman et al. 2023), additional healthcare costs (Grosse et al. 2021) and reduced employment flexibility for caregivers (Ou et al. 2015). These household‐level pressures mean that food insecurity may be more persistent, more difficult to mitigate and more strongly intertwined with daily behavioural challenges compared to non‐autistic households (Strauss et al. 2024).

Our results are consistent with a larger body of research showing that families of children with developmental disabilities are more likely to experience food insecurity (Karpur et al. 2021). The included studies highlight that families of children with ASC encounter not only economic constraints but also structural barriers, such as limited access to benefits, underemployment and low social support that contribute to material hardship (Anderson et al. 2024). The financial and emotional stress that caregivers endure is exacerbated by these larger social determinants, particularly when juggling the intricate and frequently expensive requirements related to ASC.

Recent policy shifts have also influenced food insecurity risk both in the United States and the United Kingdom. In the United States, reductions in the availability of SNAP/WIC benefits under legislative changes such as the One Big Beautiful Bill have disproportionately impacted low‐income and disabled households, including families with autistic children who are already navigating elevated caregiving and healthcare‐related expenses (USDA 2025). In the UK, proposed reforms to disability‐related benefits, including changes to eligibility for Personal Independence Payment and health‐related elements of Universal Credit, have raised concerns among disability and welfare organisations that autistic individuals and their families may experience income loss and increased material hardship (Citizens Advice 2025; Joseph Rowntree Foundation 2025). The British Dietetic Association has highlighted persistent health and nutritional inequalities faced by autistic people, reinforcing the importance of adequate social protection in mitigating these risks (BDA 2025).

Another important area where ASC and food insecurity intersect is feeding behaviours. Narrative synthesis of the included studies found that selective eating, brand‐specific preferences and sensory sensitivities common in ASC can make problems in food‐insecure households worse, despite the fact that limited data prevented meta‐analysis of this relationship. When families depend on food banks or inexpensive options that might not suit the child's needs or preferences, this becomes especially problematic (Loughborough University 2023). Additionally, research to date indicates that food parcels frequently fall short in terms of nutritional value and do not meet dietary needs related to health, culture or individual needs (Oldroyd et al. 2022; Family Fund 2023). This mismatch can lead to nutritional deficiencies, lower food acceptability and increased caregiver stress for families with autistic children.

The COVID‐19 pandemic further intensified these challenges. Multiple studies in this review found that lockdowns disrupted food access, worsened selective eating behaviours and increased caregiver distress, amplifying existing vulnerabilities (Dhillon‐Burrows et al. 2023; Panjwani et al. 2021a, 2021b). These findings underline the importance of adaptive, resilient food support systems that can respond to crisis contexts while meeting the nuanced needs of neurodiverse families (Melchior et al. 2014; Tarasuk and Vogt 2014). It is also important to note that emergency expansions of food benefits in the United States (e.g., increased SNAP allotments and pandemic‐electronic benefit transfer provisions) helped stabilise food insecurity rates for many families early in the pandemic, even as other stressors intensified (Austin and Sokol 2024). In the UK, temporary emergency measures also aimed to protect low‐income households during the pandemic. These included the Holiday Activities and Food (HAF) programme, which provided free healthy meals and childcare during school holidays to families receiving income‐related benefits, helping buffer food insecurity when regular school meals were unavailable (Department for Education 2025). Additionally, early in the pandemic, the COVID Winter Grant Scheme allocated targeted funding to local authorities to support vulnerable households with essential costs, including food, through vouchers or direct assistance (Department for Work and Pensions 2021). Food insecurity has been linked to increased social isolation, depression and family stress in addition to negative nutritional effects (Bateson et al. 2025; Ejiohuo et al. 2024). However, food‐related programs like breakfast clubs, communal kitchens and social eating programs may have two purposes: they can increase access to food while also promoting mental health and social inclusion (Blake and Cromwell 2022). Particularly for families of children with ASC, these pathways offer promising community‐level interventions that merit more investigation.

The findings of this review have implications for policy, practice and future research. The elevated prevalence of food insecurity among autistic households indicates that mainstream food aid systems are unlikely to meet families' needs if they focus solely on financial assistance. Instead, interventions must incorporate an understanding of autism‐specific sensory profiles, brand or texture‐based food preferences and the need for routine and predictability in meals. Tailored food support programs, such as sensory‐inclusive food parcels, specialised food vouchers, or dietetic services integrated within autism care pathways, may better support nutritional adequacy and reduce caregiver stress. Additionally, the methodological limitations identified, particularly inconsistent measurement tools and the dominance of US‐based data, emphasise the need for robust, standardised and internationally comparative research. Developing validated tools that capture both material hardship and autism‐specific food‐related needs would improve the accuracy and utility of future prevalence estimates.

Finally, it becomes clear that food insecurity encompasses more than just financial limitations when the definition is broadened beyond merely economic constraints. The broadest definition of food insecurity states that people are not regularly able to obtain enough safe and nourishing food for normal growth and development as well as an active, healthy life (Coleman‐Jensen et al. 2014). As a result, a diet that excludes or restricts certain food groups is a type of food insecurity because it does not provide enough nutrients. Further research is needed into particular deficiencies that are more likely to affect autistic children who are food insecure. Investigating this could yield important information about the dietary difficulties experienced by children with ASC and guide focused interventions to meet their individual requirements.

## 5 Study Limitations

The main limitation of this review was the high publication bias in the meta‐analysis, consistent with trends in public health research. Ayorinde et al. (2020) highlighted that public health reviews often suffer from unpublished or selectively published studies, especially those with non‐significant results. Small sample sizes further contributed to this bias. Despite mitigation efforts like comprehensive search strategies and a sensitivity analysis, the incomplete retrieval of studies remains a possibility. Another limitation was the variability in measurement tools for food insecurity across studies, introducing heterogeneity and affecting result comparability. Despite standardised inclusion criteria, diverse assessment methods led to inconsistencies, limiting direct comparisons and requiring cautious interpretation. Lastly, most studies used self‐reported ASC diagnosis, which may compromise diagnostic validity and lead to misclassification bias, and samples were heavily male‐dominated, reflecting longstanding diagnostic biases that may underrepresent autistic girls due to gendered symptom presentation and masking behaviours.

## 6 Conclusions

In conclusion, this systematic review provides comprehensive insights into the prevalence of food insecurity among children with ASC and their associated challenges. The meta‐analysis conducted in this review revealed a considerable prevalence of food insecurity among children with ASC, albeit based on a small evidence base. Moreover, the review identified a need for further research and tailored interventions to support children with ASC experiencing food insecurity. Efforts to standardise measurement tools within the field are also warranted to enhance the reliability and comparability of future studies.

Additionally, this review found that exploring the links between food issues, food insecurity and the social dynamics of mealtimes, as well as their potential long‐term consequences, presents an essential avenue for future research. Moreover, this review emphasises the need for tailored interventions and support mechanisms to address the complex challenges faced by children with ASC and their families, particularly in the context of food insecurity.

## Author Contributions

Conceptualization was undertaken by M.Y., E.L.G. and J.S. Methodology and validation were contributed by M.Y., E.L.G., J.S., E.T. and B.H. Formal analysis was conducted by M.Y., J.S., E.T. and B.H. Investigation involved M.Y., E.L.G., J.S., E.T. and B.H. Resources, data curation and visualisation were undertaken by M.Y., E.T. and B.H. Writing of the original draft was led by M.Y., E.T. and B.H., with review and editing contributed by all authors. Supervision and project administration were provided by E.L.G. and J.S.

## Funding

The authors have nothing to report.

## Conflicts of Interest

The authors declare no conflicts of interest.

## Supporting information


**Appendix S1:** nbu70047‐sup‐0001‐AppendixS1.docx.

## Data Availability

The data that support the findings of this study are available from the corresponding author upon reasonable request.
