# Supplementary material for: Food Insecurity in Families With Children or Young People With Autism: A Systematic Review and Meta‐Analysis
Source: Nutr Bull. 2026 Feb 17;51(2):185–99. doi: 10.1111/nbu.70047 (PMC13254693; doi:10.1111/nbu.70047)
Supplement: Supplementary file 1 — Appendix S1: nbu70047‐sup‐0001‐AppendixS1.docx. [file NBU-51-185-s001.docx]

**Supplementary File 1. Search Terms**

| **P**opulation | Autistic children and young people below the age of 25 years old and their family members | “autism” OR “autistic” OR “ASD” OR “autism spectrum disorder” OR “autism spectrum” OR “autistic disorder” AND “children” OR “young people” OR “young” OR “child*” OR “kid*” OR “youth*” OR “minor*” OR “infant*” AND “family” OR “family member*” OR “mother” OR “mum” OR “mam” OR “mom” OR “father” OR “dad” OR “siblings” OR “brother” OR “sister” |
| --- | --- | --- |
| **I**ntervention/exposure | Food insecurity | “Food insecur*” OR “Food poverty” OR “Food deprivation” OR “Food insufficiency” OR “lack of food” OR “Food hardship” |
| **C**omparators | Typically developed children | “typically developed children” OR “TDC” OR “non-ASD” OR “non-autistic” OR “typically developed” OR “TD” |
| **O**utcomes | Experiences of food insecurity and/or feeding behaviours described in cross-sectional, cohort, or quantitative research | “experience*” OR “cross-sectional” OR “cohort” OR “quantitative” OR “qualitative” OR “intervention” |
